# Mild Dissonance Preferred Over Consonance in Single Chord Perception

**DOI:** 10.1177/2041669516655812

**Published:** 2016-06-27

**Authors:** Imre Lahdelma, Tuomas Eerola

**Affiliations:** University of Jyväskylä, Finland; University of Washington, USA; Durham University, UK

**Keywords:** chord, vertical harmony, consonance/dissonance, psychoacoustics, preference

## Abstract

Previous research on harmony perception has mainly been concerned with horizontal aspects of harmony, turning less attention to how listeners perceive psychoacoustic qualities and emotions in single isolated chords. A recent study found mild dissonances to be more preferred than consonances in single chord perception, although the authors did not systematically vary register and consonance in their study; these omissions were explored here. An online empirical experiment was conducted where participants (*N* = 410) evaluated chords on the dimensions of Valence, Tension, Energy, Consonance, and Preference; 15 different chords were played with piano timbre across two octaves. The results suggest significant differences on all dimensions across chord types, and a strong correlation between perceived dissonance and tension. The register and inversions contributed to the evaluations significantly, nonmusicians distinguishing between triadic inversions similarly to musicians. The mildly dissonant minor ninth, major ninth, and minor seventh chords were rated highest for preference, regardless of musical sophistication. The role of theoretical explanations such as aggregate dyadic consonance, the inverted-U hypothesis, and psychoacoustic roughness, harmonicity, and sharpness will be discussed to account for the preference of mild dissonance over consonance in single chord perception.

## Introduction

Research on harmony perception has predominantly been concerned with harmony’s *horizontal* dimension in the form of harmonic progressions (e.g., Bigand & Parncutt, 1999; Bigand, Parncutt & Lerdahl, 1996; Sloboda, 1991; Webster & Weir, 2005). Considerably less attention has been turned to harmony’s *vertical* dimension, that is, how listeners perceive single chords (three or more simultaneous pitches) isolated from all musical context. As Huron (1994) notes: “little is known about the perception of three or more concurrent pitches  …” (p. 304) and Gabrielsson and Lindström (2010) concur: “… there is practically nothing on how different kinds of chords … may affect expression” (p. 393).

While there have been some empirical studies conducted on specifically vertical harmony perception, the emphasis has been mainly on harmonic intervals (e.g., Costa, Bitti, & Bonfiglioli, 2000; Krantz, Merker, & Madison, 2004; Maher, 1980; Oelmann & Laeng, 2009), on the major or minor triad distinction (e.g., Bakker & Martin, 2015; Crowder, 1985; Heinlein, 1928; Kastner & Crowder, 1990), or on the perception of consonance or dissonance mostly in triads (e.g., Bidelman & Krishnan, 2011; Cook, 1999; Pallesen et al., 2005; Roberts, 1986). Research addressing the perception of chords containing more than three pitches has been rare: Studies focusing directly on single chord perception while using a more diverse chord palette have been conducted by Minati et al. (2009) who applied tetrads in a neurological experiment on consonance or dissonance perception; Kuusi (2010, 2011) who investigated how listeners perceive nontraditional (nontonal) chords; and by Lahdelma and Eerola (2016) who focused exclusively on the emotion perception of single chords, spanning both triads and seventh chords.

As Parncutt (1989) puts it, “musical sounds are consonant if they are perceived to ‘sound well’ with each other (*con sonare*), and suggests that “chords are consonant if they contain no dissonant intervals” (p. 56). Consonance or dissonance can be divided into two subcategories: Single isolated intervals and chords represent *sensory consonance or dissonance* (psychoacoustics), while consonance or dissonance in chords and intervals while being part of a musical context is referred to as *musical consonance or dissonance* or *musical acoustics* (e.g., Krumhansl, 1990; Terhardt, 1984; Zwicker & Fastl, 1990).

It is widely held that sensory dissonance arises from the beating of frequency components (e.g., Hutchinson & Knopoff, 1978; Kameoka & Kuriyagawa, 1969). According to McDermott, Lehr, and Oxenham (2010) “beating occurs whenever two sinusoids of differing frequency are combined” (p. 1), which in turn creates the sound quality of *roughness* that listeners typically perceive as unpleasant. The effect of roughness is seen as prevalent in dissonant, but not in consonant musical chords (e.g., Hutchinson & Knopoff, 1978; Plomp & Levelt, 1965). Zentner and Kagan (1998) propose that the preferential bias for consonance could be innate, basing this view on their finding that infants are biologically prepared to treat consonance as more pleasant than dissonance; this view of an innate preference for consonance, however, has later been challenged by Plantinga and Trehub (2014).

Zwicker and Fastl (1990, p. 313) point out three actual factors that contribute to sensory consonance or dissonance: (a) *roughness*, (b) *sharpness*, and (c) *tonalness* (in contrast to noisiness). More recent studies (Cousineau, McDermott, & Peretz, 2012; McDermott et al., 2010), however, suggest that *harmonicity*, that is, “the extent that the sonority’s audible spectrum corresponds to a harmonic series” (Parncutt, 2014a, p. 972) plays also an important role in the perception of consonance or dissonance, possibly an even more important one than roughness. According to this view, “consonant chords derive their pleasantness not from the absence of beating, but rather from their similarity to single notes with harmonic spectra” (McDermott et al., 2010, p. 2). The study by McDermott et al. (2010) curiously indicates that harmonicity preferences correlate with musical expertise, suggesting that exposure to music amplifies preferences for harmonic frequencies because of their musical importance.

According to the results of Lahdelma and Eerola (2016), chords that are considered as mildly dissonant in terms of both *sensory* (i.e., chords containing dissonant intervals) as well as *musical* consonance or dissonance (see e.g., Révész, 1954) were actually the most preferred ones among a heterogeneous and big sample of listeners including both experts and inexperts: The mildly dissonant minor seventh and major seventh chords were more preferred than the consonant major and minor triads. This proposes an ostensible paradox: Consonance and preference or pleasantness are often seen as indisputably overlapping or even being completely *synonymous* in terms of harmony perception (see e.g., Bidelman & Krishnan, 2011; Cousineau et al., 2012; Tramo, Cariani, Delgutte, & Braida, 2001; Tymoczko, 2011). Parncutt (1989), however, aptly reminds that “relatively consonant sounds are not necessarily preferred to relatively dissonant sounds. If this were the case, single tones would always be preferred to chords” (p. 57). He proposes that the relationship between dissonance (complexity) and preference usually takes the form of an inverted-U curve: “for relatively low degrees of dissonance, preference increases with increasing dissonance, while for relatively high degrees, preference decreases with increasing dissonance” (p. 57).

While the inverted-U hypothesis (e.g., Berlyne, 1971) offers one possible explanation for why mild dissonance is preferred over consonance in single chord perception, it does not seem to be all-encompassing. For example, it cannot account for the fact that according to the data of Lahdelma and Eerola (2016), the dominant seventh chord (major–minor seventh) was less preferred than the major and minor triads, which according to the inverted-U hypothesis as such should be the other way around; the dominant seventh being somewhere in between the extremes of consonance and dissonance when considering a large number of possible chord sonorities (cf. Huron, 1994). Moreover, on an empirical note, Orr and Ohlsson (2005) did not find evidence for an inverted-U relation for experts in their experiment when testing the relationship between liking and complexity in musical improvisations.

Inspecting the phenomenon from a different angle, it is striking how in Huron’s (1994) *aggregate dyadic consonance* calculations (the sum of the consonances of all interval classes within a chord) the most consonant pitch set for tetrads (out of all possible four-pitch combinations) is the minor seventh chord, the major seventh chord being the third most consonant. Intriguingly, with regard to tetrachords, the highest amount of aggregate dyadic consonance is parallel with the evaluations for highest subjective preference in the data of Lahdelma and Eerola (2016). Huron (1994), however, reminds that “in the case of these three- and four-note sets, it is important to recognize that the consonance measures do not reflect the consonance of the complete set of concurrently sounding tones (such as the consonance of ‘a major triad’)” (p. 301). Despite this important caveat, the analogy between high aggregate dyadic consonance and preference in single chords is striking.

As pitch class sets comprising five and six pitches yield the most aggregate dyadic consonance in single chords (Huron, 1994), the aim of the current experiment is to broaden the chord palette of Lahdelma and Eerola (2016) to encompass not only triads and tetrachords but also penta- and hexachords containing most aggregate dyadic consonance to empirically test how these are perceived compared with one another and whether there indeed is the possibility that high aggregate dyadic consonance values predict preference in single chord perception.

In addition, the current experiment’s aim is to test whether the found differences in the perception of triadic inversions in the data of Lahdelma and Eerola (2016) hold up with randomized chord roots. In their data, with the major triad, the tendency was that the applied dimensions of *valence*, *tension*, and *energy* all exhibited a pattern of increasing ratings from root through first inversion to second inversion, on both applied timbres (piano and strings). A similar pattern also occurred for the dimensions of *interest or expectancy*, *happiness or joy*, and *liking or preference*. However, in their experiment, the chords were played only in a single octave (C4) and exclusively with C roots. Hence, the results could be due to register differences between the inversions (e.g., the major triad’s second inversion being higher in register than the root position and the first inversion), as pitch height reportedly affects actual music perception (e.g., Ilie & Thompson, 2006; Jaquet, Danuser, & Gomez, 2014). Thus, we feel that with regard to the triadic inversions, the randomization of chord roots across two octaves can tell us more about the role of register in accounting for the results of Lahdelma and Eerola (2016).

## Experiment

**Figure 1.**
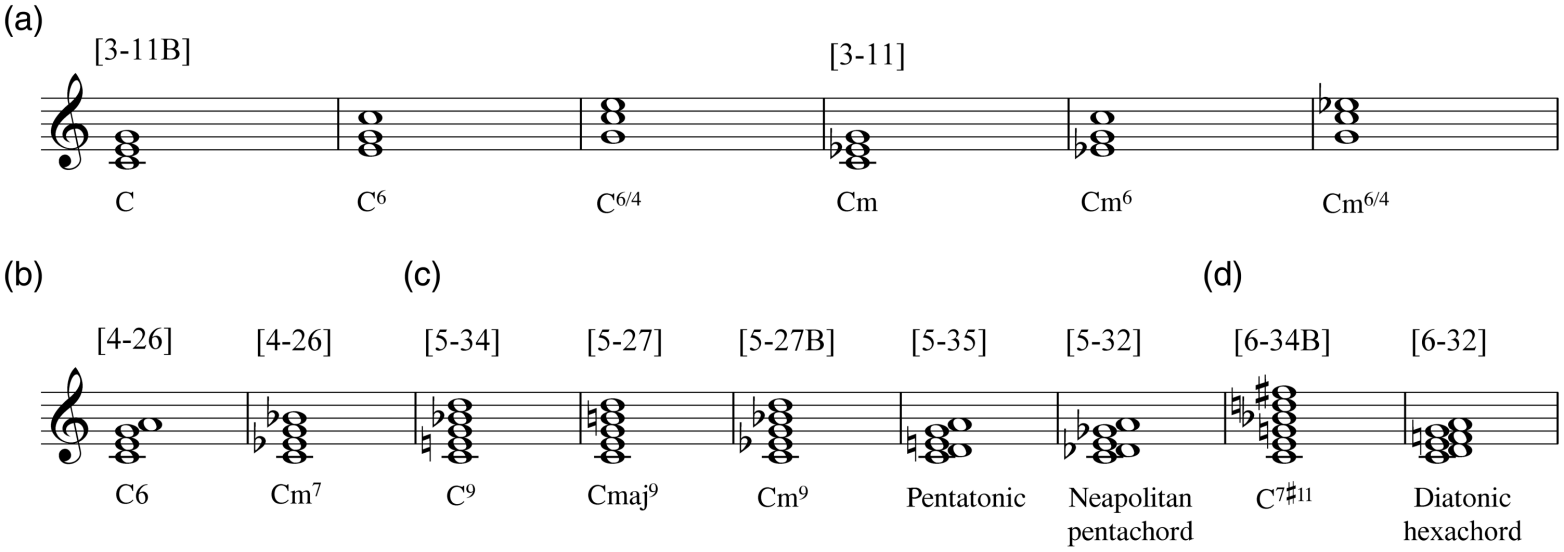
The chord stimuli. The chords are notated here with C roots; Forte pitch-class set names can be seen above each individual chord type in brackets. Additional descriptive names taken from Solomon (2005). (a) Triads with inversions, (b) Tetrachords, (c) Pentachords and (d) Hexachords.

In the current experiment, we asked participants to rate single chords (see Figure 1) isolated from all musical context (major and minor triads with inversions, selected tetra-, penta-, and hexachords in root positions) using five scales measuring separate emotional and perceptual qualities.

The first three bipolar dimensions of the five scales were adopted from Schimmack and Grob (2000). Their three-dimensional model of affect attempts to capture the core affects using the three bipolar dimensions of *valence* (intrinsic attractiveness or aversiveness), *energy arousal*, and *tension arousal*. These dimensions have been applied in studies using actual music as stimuli (e.g., Ilie & Thompson, 2006), effectively separating the effects of register on perception, as well as in an experiment conducted directly on single chord perception (Lahdelma & Eerola, 2016). The fourth applied dimension was *consonance* (used in studies by e.g., Bidelman & Krishnan, 2011; Roberts, 1986) in order to capture the participants’ subjective perception of consonance and dissonance in a given chord. The fifth dimension measured the participants’ subjective *preference* for each chord. These last two dimensions were chosen in order to investigate the amount of intersection between perceived consonance and preference with regard to single chord perception.

### Method

#### Participants

The participants for the study were recruited through the Internet with the aim of drawing the attention of both musicians and nonmusicians in order to have a substantial, heterogeneous, and international participant pool (see e.g., Honing & Ladinig, 2008 and Honing & Reips, 2008 for a review of the benefits of this strategy). The experiment was advertised in the social media (Twitter, Facebook, and LinkedIn) and on the mailing lists of different universities, music institutions, and music research communities around the world. As an incentive, three €30 gift cards to *Amazon* were drawn between all participants who left their e-mail addresses for this purpose after taking the experiment. The total amount of participants was 434, out of which 418 were considered valid for further statistical analysis; 12 participants were removed due to technical problems evident on the basis of the interface’s result database (and in most cases corroborated by reports that certain chords did not play because of internet connection problems), and 4 participants were annulled as their answers were obviously malicious in nature (i.e., no chord evaluations done or evidently random clicking in a minimum amount of time spent on the experiment website). Out of the remaining 418 participants, 8 extreme outliers were removed (described in more detail in the Results), making the final number of valid cases 410.

In total, 42 different nationalities were represented in the final participant pool (continental breakdown: 61.2% Europe, 30.5% Americas, and 8.3% others). The biggest nationality groups represented were Finland (33.9%), the USA (21.2%), and Great Britain (9.8%). The participants were aged 15 to 87 years (mean = 30.7, *SD* = 13.4, 50% males). The participants’ musical sophistication was measured with the *Ollen Musical Sophistication Index* (Ollen, 2006), a 10-item questionnaire yielding a score for each participants’ musical sophistication between 0 and 999 (mean = 545, *SD* = 336.9), the score of 500 being the threshold between a respondent being *more musically sophisticated* and *less musically sophisticated* (see Marcs Auditory Laboratories, http://marcs-survey.uws.edu.au/OMSI/omsi.php).

#### Stimuli

The chord material (Figure 1) consisted of major and minor triads (played in their root positions and in their first and second inversions, respectively), tetrachords (major sixth and minor seventh), pentachords (dominant ninth, minor ninth, major ninth, pentatonic, and Neapolitan pentachord [as referred to in Solomon, 2005]), and hexachords (dominant seventh sharp eleventh and diatonic hexachord). Only the triad chords were played with inversions in order to further investigate the results of Lahdelma and Eerola (2016), all other chords were played exclusively in their root positions. All chords were played in close position. The tetrachords, pentachords, and hexachords were selected on the basis of Huron’s (1994) table for chords containing most aggregate dyadic consonance.

As *familiarity* is an important component in chord perception (see e.g., Parncutt & Hair, 2011), we decided to include highly consonant chords (selected from a list by Tymoczko, 2011, p. 63) that are more familiar from actual musical context when compared with some of the rarer chord sonorities containing high aggregate dyadic consonance in order to see how this possibly affects the chord evaluations. The major sixth and the minor seventh represent the same pitch-class set as they contain the same pitches in different orderings; we decided to include both chords in order to investigate if there is any difference in how they are perceived depending on the chord’s root.

All selected chords were transposed with a randomization across two octaves ( ± 5 semitones around C4 and C5, the possible chord roots being all equally likely to occur within this range). Thus, the stimuli consisted of 15 chords (Figure 1) performed with piano timbre across two octaves, making the total sum of chords for each participant 30. All chords were exactly 4.8 seconds in length and played in equal temperament. The chords were generated with *Ableton Live 9* (a commercial music sequencer software), using the *Synthogy Ivory Grand Pianos II* plug-in. The applied sound font was *Steinway D Concert Grand* with a touch of ambience reverb added to the chord samples to make them sound more natural. The attack, articulation, and reverb values of the chords were kept as neutral as possible to keep the participants’ attention exclusively on the actual chords. The stimuli can be found online at http://dx.doi.org/10.7910/DVN/GE5PPL

#### Procedure

The web-based chord evaluation application was programmed with JavaScript. The application was made specifically for the purpose of the current experiment and was accessible online between May 15, 2015 and June 12, 2015. It was programmed to gather the participants’ demographic background information (gender, nationality, age), musical preference (*Short Test Of Music Preferences*; Rentfrow & Gosling, 2003), musical sophistication (*Ollen Musical Sophistication Index*; Ollen, 2006), and the type of audio device used to take the experiment.

The participants received the following instructions:

In the experiment you will be asked to rate 30 chords on 5 dimensions, and to provide some background information concerning your musical education. You can listen to each chord as many times as you like before evaluating it. Each chord should be evaluated as a separate entity, regardless of preceding or sequential chords.

The participants were asked to rate each chord on the presented 5-item scale (Appendix). The five dimensions were rated on a Likert scale ranging from 1 to 7. With *valence*, the bipolar extremes were 1 = *negative* and 7 = *positive*. With *tension*, the extremes were 1 = *relaxed* and 7 = *tense*, and with *energy*, the extremes were 1 = *low* and 7 = *high*. With *consonance*, the extremes were 1 = *rough* and 7 = *smooth*, these two poles having been used extensively in research literature (e.g., Bregman, 1994; Parncutt & Hair, 2011; van de Geer, Levelt, & Plomp, 1962). For *preference*, the applied poles were 1 = *low* and 7 = *high*. The participants were given the chance to listen each chord as many times as they wished. The ordering of the chords, the chords’ roots (across two octaves), as well as the ordering of the five dimensions were randomized for each participant.

## Results

All extreme outliers (over ± 3.0 *SD*'s in dimension aggregations, 8 sets of answers altogether) were removed from the participant pool (*N* = 418), making the final number of valid cases 410. The rating scales' internal consistency was measured with Cronbach’s alpha (range .80 − .87, see Table 1 for details). Correlations between the five variables were calculated (Table 1). The strongest correlations were found between the dimensions of *tension* and *consonance* (−.97), *tension* and *preference* (−.79), and between *tension* and *energy* (.78).

**Table 1.** Correlations Between the Rating Scales Across Chords and Register.

	Valence	Tension	Energy	Preference	Consistency
Valence					.799
Tension	−.778**				.810
Energy	−.273	.782**			.831
Preference	.631*	−.786**	−.672**		.868
Consonance	.715***	−.965**	−.724**	.715**	.844

*Note. df* = 20. Consistency refers to Cronbach's alphas.

* *p* < .01. **p < .001.

While there is certainly overlap between the dimensions of *valence*, *consonance*, and *preference*, this overlap is not complete (cf. the virtually complete negative correlation between *tension* and *consonance*) and shows that perceived consonance does not automatically result in more perceived valence and preference in single chord perception.

### The Effect of Musical Factors on the Chord Evaluations

To estimate whether the ratings across the chords and register exhibited any differences, a two-way repeated-measures analysis of variance was carried out for all five dimensions with the Chord Type and Register (Low and High) as the two within-subject factors. Chord Type consisted of the 11 main categories of chords in which the triadic inversions were collapsed into the main types of triad chords.

#### Chord Type and Register

**Figure 2.**
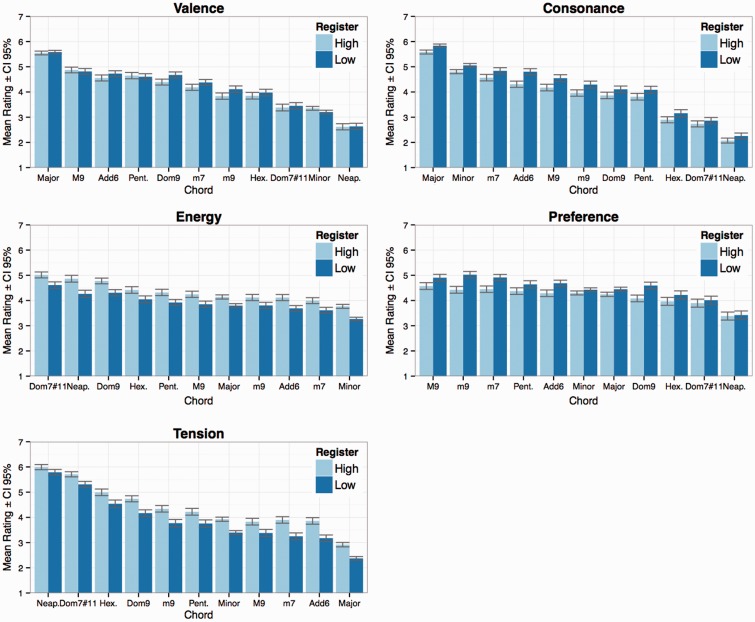
Mean ratings of the five dimensions across Chord Type and Register.

As displayed in Table 2, all scales display significant main effects of Chord Type and Register. Out of these two factors, Chord Type is typically larger, with effect sizes ranging from 0.07 to 0.37, whereas Register exhibits considerably lower effect sizes (0.001–0.04). In all cases, except for *energy*, there was also a weak interaction between the factors. The summary of the analysis of variance (Table 2) reveals that for *valence*, *tension*, and *consonance*, the differences in ratings across Chord Type were strikingly large (i.e., effect sizes above .25, which display generalized eta squared values, $\eta_{\text{G}}^{2}$, at the Chord Type column’s right side). The effect sizes across Register (the Register column’s right side) were considerably smaller, the two largest being on the dimensions of *tension* (.04) and *energy* (.03). The effect size for *valence* was conspicuously small, and these findings are in line with Parncutt (2014b) who proposes that pitch height in music is normally associated with *arousal*, not valence. Parncutt also suggests that music with a high average pitch tends to contain more energy than music with a low average pitch; this seems to hold true also for single chord perception. Ilie and Thompson (2006) also found tension to grow with pitch height in an empirical setting using actual musical excerpts as stimuli; again, this same effect is present in single chord perception as well (Figure 2).

**Table 2.** Two-Way ANOVA for All Dimensions.

	Chord Type		Register		Chord Type × Register	
	*F*	$\eta_{\text{G}}^{2}$	*F*	$\eta_{\text{G}}^{2}$	*F*	$\eta_{\text{G}}^{2}$
Valence	348.55***	.29	9.20**	.001	4.44***	.003
Tension	433.24***	.33	228.58***	.04	3.19***	.002
Energy	78.87***	.08	155.91***	.03	1.28	.0009
Consonance	538.61***	.37	77.50***	.01	1.96*	.001
Preference	65.08***	.07	86.51***	.01	6.09***	.004

*Note. df* = 10,4090 for chord, *df* = 1,409 for register, and *df* = 10,4090 for Chord × Register. All *p* values corrected for sphericity with Greenhouse-Geisser procedure and for multiple testing with Bonferroni adjustment.

* *p* < .05. ** *p* < .01. *** *p* < .001 (two-tailed).

The main effects of Chord Type across the dimensions provide further interesting results. When we look at the effects of Chord Type (aggregated over register), the perceived *valence* was highest for the major triad with a mean of 5.56 (*SD* = 1.27) and lowest for the Neapolitan pentachord with a mean of 2.62 (*SD* = 1.26). Perceived *tension* was highest for the Neapolitan pentachord with a mean of 5.89 (*SD* = 1.11), followed by the dominant seventh sharp eleventh chord with a mean of 5.51 (*SD* = 1.15). The lowest mean rating on the dimension of *tension* was for the major triad’s mean of 2.65 (*SD* = 1.46). Perceived *energy* was highest for the dominant seventh sharp eleventh chord with a mean of 4.81 (*SD* = 1.28), followed by the Neapolitan pentachord’s mean of 4.56 (*SD* = 1.47), and the dominant ninth’s mean of 4.54 (*SD* = 1.25). The lowest mean rating on the dimension of *energy* was for the minor triad with a mean of 3.52 (*SD* = 1.34). The perceived *consonance* mean rating was highest for the major triad with a mean of 5.71 (*SD* = 1.31), the lowest for the Neapolitan pentachord’s mean of 2.16 (*SD* = 1.13). On a side note, it is worth noting how the added sixth chord (*M* = 4.49, *SD* = 1.31) was perceived as less consonant than the minor seventh chord (*M* = 4.68, *SD* = 1.30), even though the chords represent the same pitch class set. The highest rating on the dimension *preference* was for the major ninth chord with a mean of 4.74 (*SD* = 1.39), followed closely by the minor ninth’s mean of 4.72 (*SD* = 1.39), and the minor seventh’s mean of 4.68 (*SD* = 1.30). The lowest mean rating on the dimension of *preference* was for the Neapolitan pentachord with a mean of 3.40 (*SD* = 1.65), followed by the dominant seventh sharp eleventh chord’s mean of 3.95 (*SD* = 1.65).

We also explored the influence of additional variables such as diatonicity (the proportion of tones belonging to diatonic scales within each chord) and chord ambitus (difference between highest and lowest tone in semitones) to ANOVA analyses as within-subject covariates, but both of these variables failed to make an impact on the results.

#### Triadic inversions

**Figure 3.**
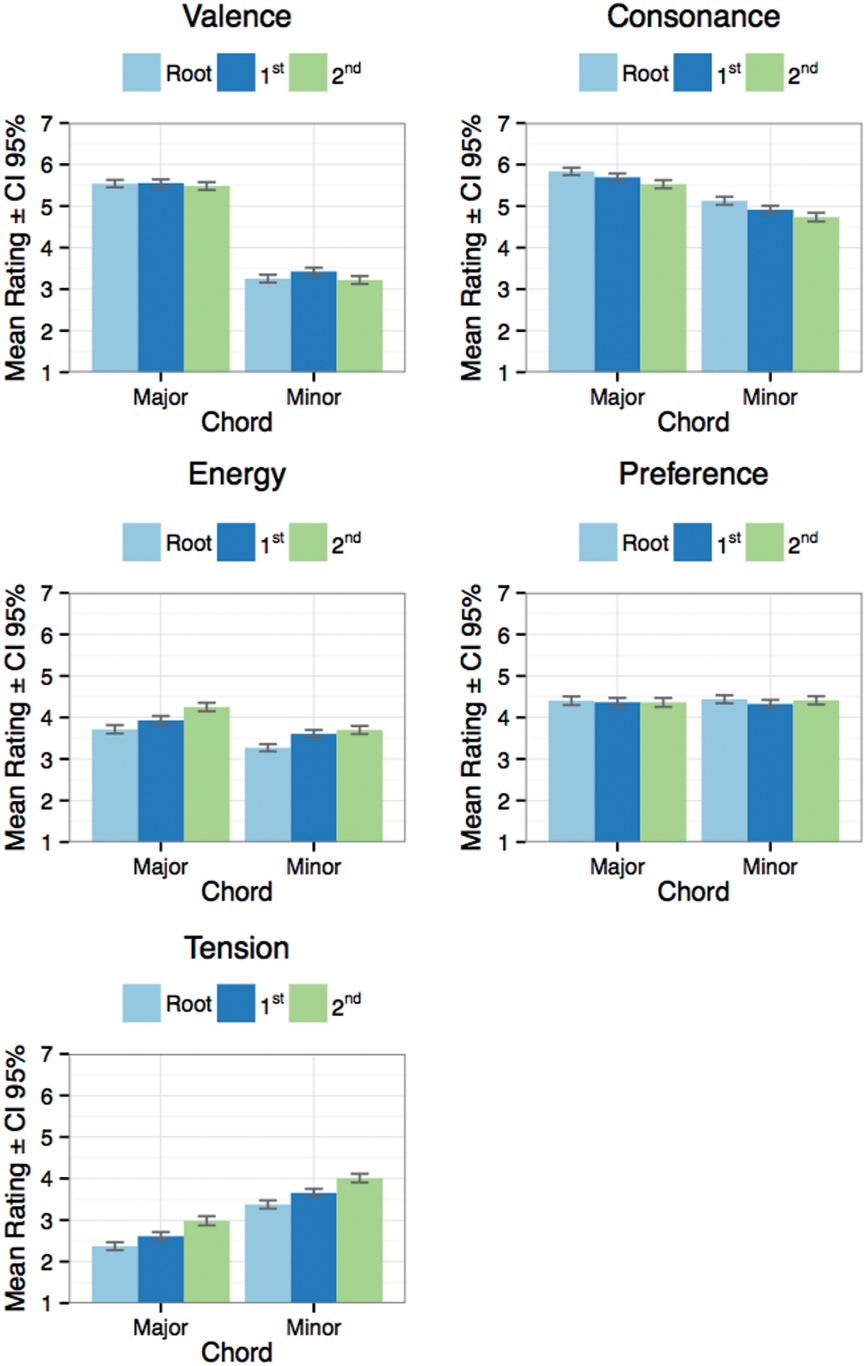
Mean ratings of the triadic inversions across all dimensions.

As can be seen from Table 3, the perception of triadic inversions is mostly in line with the results of Lahdelma and Eerola (2016): *Energy* and *tension* exhibit significant main effects with the ratings growing from root through first inversion to second inversion in both major and minor triads, and the scale of *preference* does not exhibit a significant main effect with regard to inversions. The current study’s added scale of *consonance or dissonance* exhibits a significant main effect with an opposing pattern when compared with the scales of *tension* and *energy*: In both major and minor triads, perceived consonance decreases from root through first inversion to second inversion (Figure 3). With regard to the major triad, the least amount of perceived consonance in the chords’ second inversion is notably in line with musical convention (see e.g., Randel, 2003); intriguingly, however, this pattern of perception was not influenced by musical sophistication. Strikingly, both musicians and nonmusicians distinguished between the triadic inversions on the dimensions of energy, tension, and consonance or dissonance similarly. To our knowledge, the current experiment is the first one to empirically demonstrate this trend.

**Table 3.** Two-Way ANOVA Across the Triadic Inversions for All Dimensions.

	Inversion		Chord Type		Interaction	
	*F*	$\eta_{\text{G}}^{2}$	*F*	$\eta_{\text{G}}^{2}$	*F*	$\eta_{\text{G}}^{2}$
Valence	5.99*	0.00	998.3**	0.53	3.38*	0.00
Tension	101.5**	0.04	430.1**	0.16	0.16	0.00
Energy	56.6**	0.03	79.5**	0.04	4.0*	0.00
Consonance	36.9**	0.01	325.7**	0.11	0.7	0.00
Preference	1.3	0.00	0.1	0.00	1.1	0.00

*Note. df* = 1,409 for chord, *df* = 2,818 for inversion, and *df* = 2,818 for Chord × Inversion. All *p* values corrected for sphericity with Greenhouse-Geisser procedure and for multiple testing with Bonferroni adjustment.

* *p* < .05. **p < .001 (two-tailed).

The only notable difference between the current data and the results of Lahdelma and Eerola (2016) is on the scale of *valence*. While exhibiting a statistically significant main effect, this significance is considerably smaller in the current data and showcases a different pattern. The randomization of roots seems to have dissolved virtually any perceived difference between the major triad’s inversions, and the minor triad’s first inversion was perceived as containing the most amount of positive *valence* (Figure 3); in the data of Lahdelma and Eerola (2016), the difference between the minor triad’s first and second inversions with regard to valence was negligible. However, the current finding could be explained with the fact that the minor triad’s first inversion has a major third above the bass and might hence sound somewhat “major.” Curiously, Hutchinson and Knopoff (1979) suggest that the minor triad’s first inversion is actually the most consonant of the chords’ inversions; paradoxically this was not corroborated by the current empirical data (despite the most perceived *valence* in this particular inversion), as the minor triad’s root position was perceived as containing more consonance than its first inversion.

In sum, the randomized chord roots of the triads might provide a slightly more accurate picture of the perceived differences between the triadic inversions, but the overall tendency of the current results is quite similar to the findings of Lahdelma and Eerola (2016) who did not randomize chord roots in their study and played the triads exclusively with C-roots.

### The Effect of Background Factors on the Chord Evaluations

Past studies indicate that gender (e.g., Costa et al., 2000; Lahdelma & Eerola, 2016) and musical expertise (e.g., Lahdelma & Eerola, 2016; McLachlan, Marco, Light, & Wilson, 2013) may affect vertical harmony perception. Moreover, it has been suggested that familiarity affects the perception of chords (e.g., McLachlan et al., 2013; Parncutt & Hair, 2011) and the perception of consonance or dissonance in general (e.g., Cazden, 1972; Heyduk, 1975).

In the current study, musical expertise was assessed with the *Ollen Musical Sophistication Index* (Ollen, 2006); music preferences were inferred from the ratings of 13 genres that were recoded into four meta-genres suggested by Rentfrow and Gosling (2003) according to hierarchical cluster analysis clustering the participants according to the similarity of their music preferences into four clusters. Each participant belonged to one of these clusters, labeled as *Reflective or Complex* (*n* = 215), *Intense or Rebellious* (*n* = 86), *Upbeat or Conventional* (*n* = 76), or *Energetic or Rhythmic* (*n* = 33). Separate mixed ANOVAs were carried out with emotion ratings across the three between-subjects factors (Gender, Musical Expertise, and Music Preferences) reported in Table 4.

**Table 4.** ANOVA Summary for All Ratings Across the Main Background Variables.

	Gender		Musical expertise		Music preferences	
	*F*	$\eta_{\text{G}}^{2}$	*F*	$\eta_{\text{G}}^{2}$	*F*	$\eta_{\text{G}}^{2}$
Valence	7.21*	0.00	12.86*	0.01	3.84*	0.01
Energy	0.05	0.00	2.42	0.00	3.22*	0.00
Tension	0.85	0.00	0.18	0.00	0.52	0.00
Consonance	5.6*	0.00	5.95*	0.01	0.91	0.00
Preference	7.3*	0.01	8.64*	0.01	3.6*	0.01

* *p* < .05.

Most of the ratings scales did not yield significant main effects; we will now briefly outline the ones that actually portrayed differences. For *valence* ratings, Gender, Musical Expertise, and Music Preferences yielded significant differences; males rated the chords as more positively valenced (*M* = 4.30, *SD* = 1.53) than females (*M* = 4.15, *SD* = 1.60), musicians more positively (*M* = 4.36, *SD* = 1.57) than nonmusicians (*M* = 4.07, *SD* = 1.54), and those labeled as preferring music that is “reflective and complex” also had higher ratings of valence (*M* = 4.33, *SD* = 1.57) than the other three music preference groups (*M* = 4.11, *SD* = 1.52). For ratings of *energy*, only Music Preferences showed significant differences (those classified as listening to Upbeat or Conventional music, *M* = 4.09, *SD* = 1.39, whereas listeners of Intense or Rebellious music rated the chords lower on energy, *M* = 3.89, *SD* = 1.33). With respect to *tension*, none of the background variables contributed to the chord ratings. *Consonance*, on the other hand, exhibited differences according to Gender and Musical Expertise, where males (*M* = 4.42, *SD* = 1.66) displayed higher ratings than females (*M* = 4.32, *SD* = 1.71) and those listening to Reflective or Complex music displayed higher ratings (*M* = 4.44, *SD* = 1.73) than other listeners (*M* = 4.24, *SD* = 1.62). Finally, *preference* indicated significant differences across the background variables, being very similar to the pattern exhibited by *valence* (males, musicians, and those preferring reflective and complex music showing higher ratings of preference for all chords). It is important to note here that few of the scales displayed interactions between background variables and chords. The exceptions were *energy*, where Musical Expertise displayed an interaction with chord types, *F*(10, 3940) = 3.74, *p* < .001, and *tension*, where Musical Expertise, *F*(10, 3940) = 6.37, *p* < .001, and Gender, *F* = 1.83, *p* < .001, interacted with chord types. Also *consonance* interacted with Musical Expertise and chords (*F* = 3.51, *p* < .001), as well as *preference*, where Music Preferences created an interaction with the chord types (*F* = 2.28, *p* < .001).

To summarize, these results suggest that those with higher musical expertise perceived the chords as more positive in valence, more consonant, and also preferred the chords more. This finding is in line with the notion that familiarity in fact affects the perception of chords (McLachlan et al., 2013), as well as the perception of consonance or dissonance in general (Cazden, 1972; Heyduk, 1975). In the context of all ratings, the magnitude of variations according to background, however, is considerably small and negligible (effect sizes < .01).

### Acoustic Properties of the Chords

**Figure 4.**
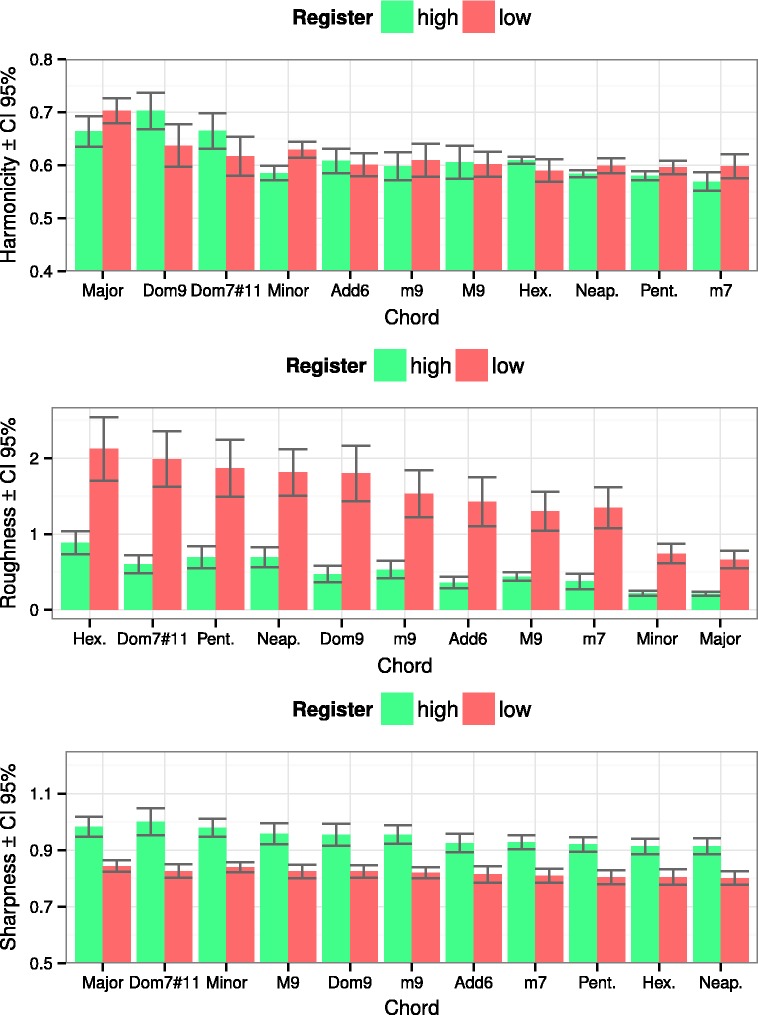
Harmonicity, Roughness, and Sharpness values across Chord Types and Register.

To examine the relationship between psychoacoustic properties and perceptual evaluations of the chords, few selected features were extracted using MIR toolbox (version 1.6.1; Lartillot, Toiviainen, & Eerola, 2008) and custom MATLAB functions based on prior studies. These were (a) harmonicity (Jensen, 1999) that accounts for the regularity of the amplitude of adjoining partials, (b) roughness that captures the sensory beating of the partials in the sound using a psychoacoustic model by Vassilakis (2001), and (c) sharpness that is related to the high-frequency content of the sound (Zwicker & Fastl, 1991). Figure 4 displays the mean values across the chord types for all three features. Moreover, to formally connect the ratings to these descriptors, a linear regression was used to assess the degree of fit between the descriptors and the ratings (see Table 5).

**Table 5.** Regression Results With Harmonicity, Roughness, and Sharpness Across the Mean Ratings for All Chords (Triadic Inversions Collapsed Into Main Chord).

	Harmonicity β	Roughness β	Sharpness β	*R^2^*	*F*	*p*
Valence	0.341	−0.476	−0.470	0.187	1.38	0.282
Tension	−0.142	1.230**	1.257 **	0.447	4.84	0.012
Energy	0.211	1.232***	1.481 ***	0.603	9.13	0.001
Consonance	0.209	−1.267***	−1.134***	0.475	5.43	0.008
Preference	−0.050	−0.582	−0.788	0.171	1.24	0.326

*Note.* Normalized betas and the model fit indices are shown.

** *p* < .01. ***p < .01. *df* = 3,18.

As can be seen in Table 5, roughness and sharpness correlate statistically significantly with the dimensions of *tension*, *energy*, and *consonance*. Both roughness and sharpness correlate positively with *tension* and especially with *energy*, while negatively with *consonance*. We suggest that the lesser amount of perceived consonance in chords played in the higher register (Figure 2) could be explained with the effect of sharpness, as chords in the lower register actually have significantly more objective roughness compared with chords in the higher register (Figure 4), despite being subjectively perceived as more consonant. This finding is in line with the notion that sharpness is another form of sensory dissonance (in addition to roughness), caused by energy at high frequencies (see Aures, 1985a, 1985b).

With regard to the difference between objective roughness and subjective dissonance, the current data offer some intriguing insights. As can be seen from Figure 4, the diatonic hexachord is theoretically more rough than the dominant sharp eleventh chord, and the pentatonic chord more rough than the Neapolitan pentachord. The ordering of these chords’ subjective dissonance, however, was exactly the opposite: the pentatonic chord was perceived as significantly more consonant compared with the Neapolitan pentachord and the diatonic hexachord slightly more consonant compared with the dominant sharp eleventh chord (Figure 2). This difference could be explained with Johnson-Laird, Kang, and Leong (2012) concept of *tonal dissonance*. They suggest that dissonance

Results from a combination of sensory and tonal dissonance, where ‘sensory’ dissonance arises … in particular from roughness (i.e., the rapid beating of partials), and ‘tonal’ dissonance is a consequence of high-level cognitive processes that rely on a tacit knowledge of the principles of tonality. (p. 24)

They propose that tonal dissonance depends on the scales in which the chords can occur: “chords occurring in a major scale should be less dissonant than chords occurring only in a minor scale, which in turn should be less dissonant than chords occurring in neither sort of scale” (p. 24). The Neapolitan pentachord is not present in either of the scales, and it was in fact perceived as the least consonant and was the least preferred of all the presented chords in the current experiment. The second least consonant and preferred dominant sharp eleventh chord is not present in a major scale (cf. the diatonic hexachord) either, but could theoretically be constructed from the melodic minor scale’s pitches in an extended tonality. The rest of the chords applied in the current experiment contain no tonal dissonance. Hence, the results clearly corroborate Johnson-Laird et al.’s (2012) theory of tonal dissonance with regard to single chord perception.

It is somewhat surprising that harmonicity did not exhibit statistically significant correlations with any of the five dimensions. As higher harmonicity is often seen as resulting in a higher amount of perceived consonance (e.g., Cousineau et al., 2012; McDermott et al., 2010), and consonance in turn being often described as a synonym for pleasantness (e.g., Bidelman & Krishnan, 2011; Bones, Hopkins, Krishnan, & Plack, 2014), the data of the current study imply that harmonicity does not automatically result in consonance and preference in the case of single isolated chords consisting of three or more pitches. Concrete examples of this phenomenon in the current data are the chords of the dominant ninth and the dominant sharp eleventh: Both chords have strikingly high harmonicity values (Figure 4) but were nonetheless rated quite low on the dimensions of *consonance* and *preference* (Figure 2). The pentatonic chord in turn has a low amount of harmonicity but was rated relatively high for *preference*. The chord with the lowest amount of harmonicity, the minor seventh, was in fact perceived as the third most consonant and also the third most preferred among the presented chords. The obtained results might also be influenced by the piano timbre that was used in the current experiment. We will return to these questions in the Discussion part.

As harmonicity, roughness, and sharpness by themselves did not correlate statistically significantly with the dimension of *preference*, we will next outline the possibility of how aggregate dyadic consonance (Huron, 1994) might affect perceived preference in single chords.

### Aggregate Dyadic Consonance

**Figure 5.**
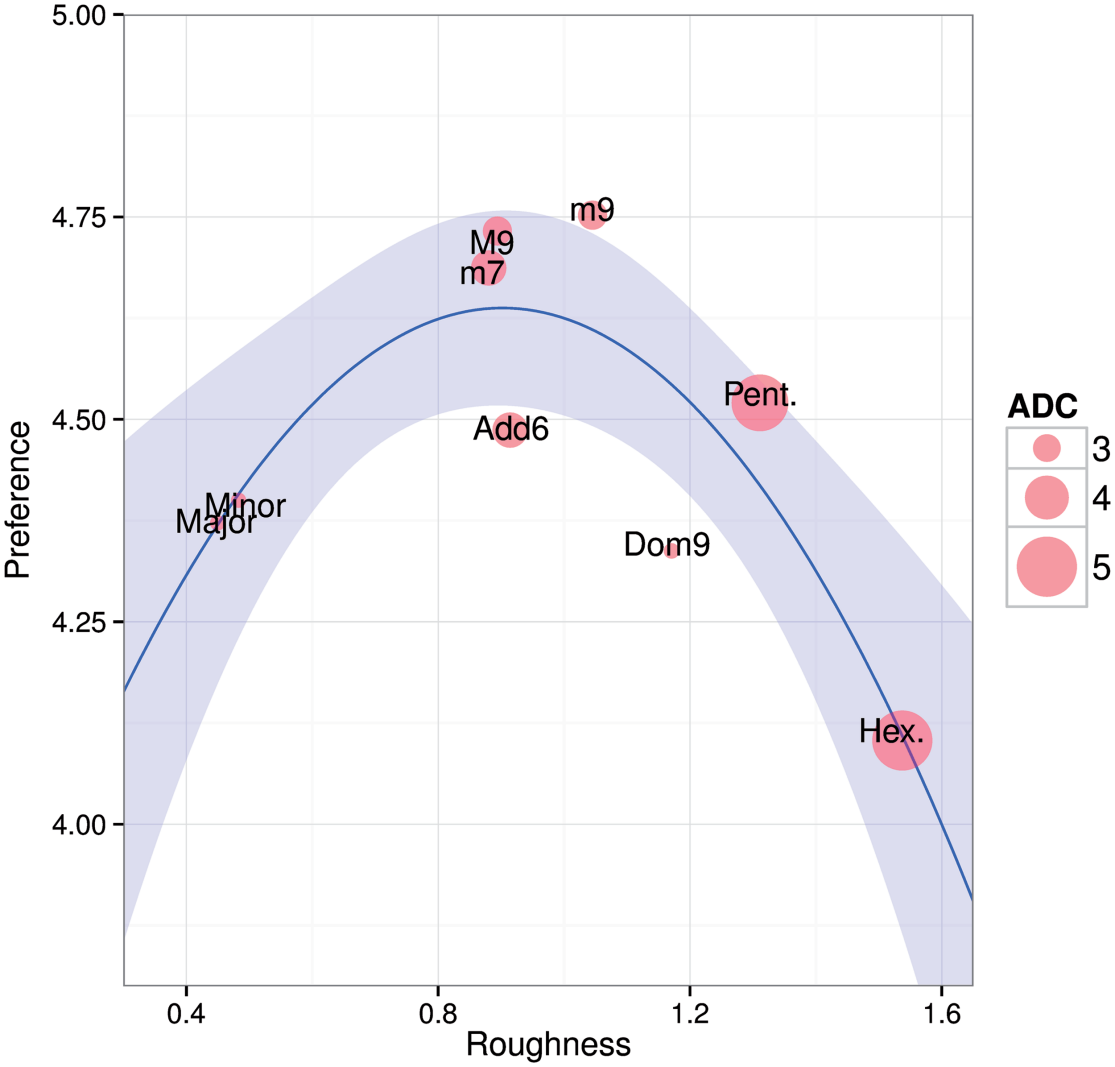
Preference, Roughness, and Aggregate Dyadic Consonance values across Chord Types, excluding chords containing tonal dissonance.

Aggregate dyadic consonance and roughness seem to be related to preference in a curvilinear fashion. There is no linear correlation between the means of the chords in terms of preference and aggregate dyadic consonance (*r* = −.16, *p* = .68) or roughness (*r* = −.24, *p* = .54), but a second-order polynominal fits the relation better (see Figure 5), especially for roughness (*R*^2adj ^= .58, *p* < .05). In other words, combining the aspects of roughness and aggregate dyadic consonance could explain why mildly dissonant chords are most preferred. The chords of the dominant sharp eleventh and the Neapolitan pentachord are omitted from this model, as they contain tonal dissonance (Johnson-Laird et al., 2012).

As can be seen from Figure 5, chords that contain a medium amount of roughness (minor ninth, minor ninth, minor seventh, added sixth, and dominant ninth) are not equally highly preferred as would be predicted by the inverted-U hypothesis. The minor ninth, the major ninth, and the minor seventh chords are more preferred than the other semi-rough chords, possibly because they contain high aggregate dyadic consonance. While the added sixth chord contains the same amount of aggregate dyadic consonance as the minor seventh chord, it however contains slightly more roughness; the major second interval present in the chord clearly has a negative impact on its preference, possibly due to enculturation (cf. Krantz et al., 2004). As for the pentatonic and hexatonic chords, these chords also contain high amounts of aggregate dyadic consonance, but the overall roughness of these chords seems to “overrule” their preference, especially in the hexatonic chord’s case. The preference ratings dramatically drop between the pentatonic and the hexatonic chords: It could be argued that there is a critical threshold of roughness in single chord perception which cannot be exceeded in order for the chord to gain high preference ratings.

Curiously, however, the high amount of roughness and subjective dissonance in the pentatonic chord should theoretically predict a much higher aversion—its high aggregate dyadic consonance might explain why the chord is relatively preferred nonetheless. In other words, a moderately high amount of roughness does not automatically result in declined preference in single chord perception. In the hexatonic chord’s case, the overall roughness of the chord seems to cross that critical threshold for roughness: this particular chord is the roughest of all the presented chords, and hence not preferred despite its theoretically high aggregate dyadic consonance. Thus, interestingly a combination of the U-theory with aggregate dyadic consonance seems to most effectively predict preference in single chord perception: the most preferred chords contain a moderate amount of aggregate dyadic consonance. It is important to keep in mind, however, that the current model has only nine data points; further research is needed to shed light on the role of aggregate dyadic consonance on the preference of single chords.

## Discussion

This study aimed to investigate how listeners perceive single chords across a 5-item scale of dimensions consisting of *valence*, *tension*, *energy*, *consonance*, and *preference*. The results suggest that mildly dissonant chords in terms of both *musical* (see e.g., Révész, 1954) and *sensory* consonance or dissonance were actually more preferred than maximally consonant chords among a large and heterogeneous pool of participants, across both expert and inexpert listeners.

We outlined theoretical explanations to account for the preference of mild dissonance in single chord perception. These include *aggregate dyadic consonance* (Huron, 1994), the inverted-U hypothesis (e.g., Berlyne, 1971), as well as the role of psychoacoustic phenomena in the form of harmonicity, roughness, and sharpness. We feel that the inverted-U hypothesis is not necessarily all encompassing to account for the preference of mild dissonances: Both the dominant seventh chord in the data of Lahdelma and Eerola (2016) as well as the dominant ninth in the current study were rated low for preference even though these chords are representing middle ground in terms of complexity when considering a wide range of sonorities.

The overall results suggest that the background factors of gender, musical sophistication, and musical preferences affected single chord evaluations to some extent—musicians interestingly objecting less to dissonance than nonmusicians. The overall magnitude of variations according to background was, however, quite small. Nonetheless, the findings are in line with propositions that familiarity affects chord perception (McLachlan et al., 2013) as well as the evaluation of consonance or dissonance (Cazden, 1972; Heyduk, 1975). On the other hand, it is intriguing how familiarity does not seem to predict preference in the dominant ninth chord’s case: It is the only pentad present in major–minor tonality and should thus be more familiar than the other, possibly more exotic five-pitch sonorities used in the current experiment. Despite its familiarity, the dominant ninth was the least preferred pentad after the Neapolitan pentachord. This is somewhat surprising taking the high amount of harmonicity and only the moderate amount of roughness present in the chord. We surmise that the low preference for the dominant seventh chord (Lahdelma & Eerola, 2016) and the current study’s dominant ninth chord might stem from the culturally loaded tritone interval present in these chords. The avoidance of tritone as an interval has both psychoacoustic and cultural origins (Parncutt, 1989, 2014a), and this avoidance seems to influence also the perception of chords in which it is present. The chord also contains low aggregate dyadic consonance. The low-perceived consonance and preference of the Neapolitan pentachord also suggests the role of enculturation in the form of *tonal dissonance* (Johnson-Laird et al., 2012) instead of a purely psychoacoustic explanation: This particular chord was the least preferred sonority and judged clearly as subjectively least consonant, despite not being among the objectively roughest chords of the presented stimuli. Hence, we see that enculturation indeed affects judgments of sensory consonance or dissonance also even in single isolated chords, not just in chords within a musical context (cf. Minati et al., 2009).

Also the difference between the perceived amount of consonance in the common pitch class set of the added sixth and the minor seventh chords is intriguing; the added sixth chord was perceived as more dissonant than the minor seventh chord. We surmise that this is caused by the slightly higher amount of roughness that the added sixth chord contains when compared with the minor seventh chord. An interesting detail is that the added sixth chord was nonetheless perceived as more positive in valence than the minor seventh chord. This could imply that the added sixth is more affiliated with the major triad because of its root when compared with the minor seventh, and again suggests that enculturation affects single chord perception in addition to psychoacoustics.

The negligible role of harmonicity in the perception of single isolated chords is somewhat surprising, especially when considering the importance it has been given in previous research (e.g., Cousineau et al., 2012; McDermott et al., 2010) with regard to the question of consonance or dissonance. However, this finding is in line with Bregman (1994), who suggests that “harmonicity may not be critical for chord perception” (p. 496). Also, according to Mellinger and Mont-Reynaud (1996) the relationship between harmonicity and the perception of harmony is a complex one, and for example, perceived pleasantness is not necessarily completely tied to harmonicity.

Parncutt (2012) proposes that dissonance is in fact based on a *combination* of roughness, harmonicity, and familiarity. If we consider chord perception encompassing also *emotion* perception, Lahdelma and Eerola (2015) demonstrate a theoretical possibility of the role of harmonicity affecting the perception of complex musical emotions conveyed by single isolated chords. With the dimensions used in the current study, however, harmonicity does not offer any significant explanation to account for the results. This may have to do also with the fact that the current experiment applied only the piano timbre. According to Pierce (1999), the small departures from perfect harmonicity are important to the piano sound; in other words, harmonicity is presumably more important in nonpercussive, steady sounds than in percussive, rapidly fading sounds. The role of timbre with regard to the importance of harmonicity in single chord perception is a crucial question and should be addressed with future experiments. Also, the question of the relationship between sensory and musical consonance is a fascinating one: How does the perception of single chords change when heard in different kinds of musical contexts (cf. Bharucha & Stoeckig, 1986; Krumhansl, 1990)?

We see that the possible role of pitch relations (aggregate dyadic consonance) with regard to the perception of single isolated chords should be examined further. The role of aggregate dyadic consonance as an explanation for why the relationship between the lack of roughness and preference is not linear could be studied with a higher number of chord sonorities. Also, the crucial threshold of maximum roughness in simultaneous pitch combinations resulting in a decline of preference should be investigated.

The current results suggest that vertical harmony perception may have more to do with horizontal harmony perception with regard to single chords than has been previously thought; this finding is in line with Tramo et al. (2001) who point out that “a listener’s implicit (or explicit) knowledge about harmony in the horizontal dimension bears on harmony perception in the vertical dimension” (p. 96). Furthermore, it is tantalizing to draw a parallel between these two distinct aspects of harmony when considering a historical point of view. As Parncutt (1989) points out (referring to Grout, 1960), horizontal intervals between tones existed before simultaneous tones in music: “History suggests … that musical intervals (octaves, fifths) between sequential tones existed long before people started singing or playing tones simultaneously in music … ” (p. 9). Could this evolution of harmony somehow still affect the perception of vertical sonorities? The question of how the ear parses the overall consonance of simultaneous intervals in vertical pitch combinations or whether it does so remains to be examined with future research.

## Appendix

### Definitions of Each Dimension on the 5-Item Scale

This is how the scales were defined and explained to the participants in the experiment.

1. Valence. Is the chord conveying positive or negative emotions?
2. Tension. How tense do you think the chord is? Is it calm and relaxed or tense and agitated?
3. Energy. Do you think the chord is strong and energetic, or weak and feeble?
4. Consonance. How smooth do you think the chord is?
5. Preference. How much did you like the chord? Note that this is a purely subjective question: for example, you may like a chord no matter how negative or harsh it sounds.
